# Study on the role of the combination of quercetin and lutein in alleviating ulcerative colitis in mice

**DOI:** 10.3389/fnut.2025.1698231

**Published:** 2025-10-17

**Authors:** Zhiyue Xu, Si Mi, Bimal Chitrakar, Liwen Wang, Yingxi Li, Renli Shi, Yaxin Sang, Wenlong Yu, Xianghong Wang

**Affiliations:** College of Food Science and Technology, Hebei Agricultural University, Baoding, China

**Keywords:** quercetin, lutein, colitis, intestinal microbiota, inflammatory factor

## Abstract

**Introduction:**

This study aims to investigate the ameliorative effects and molecular mechanisms of combined quercetin and luteolin intervention on dextran sulfate sodium (DSS)-induced colitis in mice.

**Methods:**

By establishing a DSS-induced colitis model, we systematically evaluated the comprehensive effects of the combined treatment on colonic histopathological damage, inflammatory cytokine expression, intestinal barrier function, and gut microbiota composition.

**Results:**

The quercetin-luteolin combination treatment group significantly ameliorated colon shortening, with colon length restored to 7.33 ± 0.09 cm (*p* < 0.05), reduced the severity of colonic ulcers, and decreased the disease activity index. The protective effects were manifested in two aspects: first, by synergistically upregulating the expression of tight junction proteins ZO-1, claudin-1, and occludin, the combined treatment maintained the functional integrity of the intestinal epithelial barrier; second, by inhibiting the PI3K/AKT/NF-κB signaling pathway, oxidative stress indicators were reduced by 52.6 and 20.2% (*p* < 0.05)., and the mRNA expression levels of inflammatory factors such as IL-1β, MyD88, P65, TNF-α, and TLR2 were downregulated. The expression level of AKT mRNA decreased by 69.5% (*p* < 0.05). In the LU + QR group compared to the IBD group. Additionally, the combined treatment reshaped the gut microbiota of colitis mice by modulating the relative abundances of Firmicutes, Bacteroidota, and Pseudomonadota.

**Discussion:**

Ultimately, a synergistic alleviation model characterized by “inhibition of inflammatory pathways—restoration of barrier function—regulation of microbial homeostasis” was established. This study provides a theoretical foundation for the application of quercetin and luteolin in the treatment of colitis.

## 1 Introduction

Inflammatory bowel disease (IBD) encompasses two primary subtypes: ulcerative colitis (UC) and Crohn’s disease (CD) (1). Patients with colitis frequently experience symptoms such as reduction in body weight, loose stools, abdominal pain, and hemorrhagic discharge from the rectum, which can persist over extended periods and result in significant morbidity (2). Although the pathogenesis of inflammatory bowel disease (IBD) remains incompletely understood, accumulating evidence suggests that it involves a complex interplay of genetic susceptibility, oxidative stress, intestinal microbiota dysbiosis, immune dysregulation, and environmental factors. Furthermore, the overproduction of inflammatory mediators exacerbates intestinal barrier dysfunction, leading to tissue inflammation (3). Since the early 21st century, IBD has been recognized as one of the most prevalent gastrointestinal disorders, particularly in newly industrialized nations (4). Current pharmacological treatments for IBD include aminosalicylates, glucocorticoids, immunosuppressive agents, and biologics such as anti-tumor necrosis factor therapies. However, these interventions are associated with significant side effects, including immunosuppression and an increased risk of infections when administered over prolonged periods (5). Consequently, there is an urgent need for safer, multi-targeted therapeutic agents. Natural compounds, particularly flavonoids and lutein, have emerged as promising candidates due to their inflammation-inhibiting, oxidation-inhibiting, and microbiota-modulating properties.

Quercetin, a flavonoid prevalent in various vegetables and fruits, exhibits notable anti-inflammatory, oxidation-inhibiting, and anticancer properties (6). Recent research has shown that quercetin can alleviate colitis through multiple mechanisms. In a model of colitis induced by dextran sulfate sodium (DSS), quercetin significantly reduced weight loss and mitigated signs of histological damage in mice (7). Furthermore, Topçu-Tarladaçalışır et al. (8) induced colitis via rectal administration of 2,4,6-trinitrobenzenesulfonic acid (TNBS) and observed that quercetin administration led to a reduction in colonic disease activity index. Oxidative stress levels, and inflammatory marker concentrations in mice. Oxidative stress plays a significant role in colitis, while quercetin enhances cell proliferation and increases intracellular glutathione (GSH) concentration by up-regulating the transcription of glutamate-cysteine ligase, catalytic subunit (GCLC), thereby eliminating excessive reactive oxygen species (ROS). Additionally, quercetin regulates oxidative stress by inducing an increase in extracellular hydrogen peroxide concentration through the inhibition of aquaporin 3 and the up-regulation of nicotinamide adenine dinucleotide phosphate oxidase 1/2 (NOX1/2) (7). Changes in the stable structural composition of the intestinal flora, along with an increase in the abundance of pathogenic bacteria, lead to an imbalance in intestinal flora homeostasis, which can result in intestinal diseases (9). Quercetin has been shown to improve intestinal flora and its metabolite, short-chain fatty acids, while also protecting the intestinal barrier against DSS-induced colitis in mice (10). Furthermore, the addition of quercetin to the diet of mice attenuates DSS-triggered colitis by strengthening intestinal integrity and hepatic antioxidant capacity (11). Lutein, a carotenoid abundant in green leafy vegetables and egg yolks, is recognized for its role in eye health (12). Recent research has broadened its application to gastrointestinal inflammation (13). Studies indicate that lutein reduces glutathione levels and catalase activity, normalizes superoxide dismutase and glutathione-S-transferase activities, and improves endogenous oxidative defenses in DSS-induced ulcerative colitis (UC) in mice (14). Moreover, lutein hydrogel has been found to increase the level of intestinal tight junction proteins ZO-1, claudin-1, and occluding, thereby maintaining the integrity of the intestinal barrier (3).

Xu et al. (15) demonstrated that the co-administration of baicalin and emodin synergistically attenuated DSS-induced colitis, as evidenced by a more pronounced reduction in CD14/TLR4/NF-κB pathway protein expression and a greater increase in PPAR-γ protein levels in the colon compared to monotherapies. This enhanced efficacy underscores the advantage of combinatorial phytochemical treatment over single-component interventions. Similarly, cereal and leguminous flavonoids exhibited strong anti-inflammatory synergy in LPS-stimulated colonocytes, likely through interdependent mechanistic targeting (16). These findings collectively highlight the rational basis for employing multi-component natural product combinations—such as quercetin and lutein—to achieve amplified therapeutic outcomes through complementary mechanisms.

Both lutein and quercetin have demonstrated individual efficacy in alleviating colitis. In the previous study in our laboratory, it was found that flavonoids can affect the bioavailability of lutein by regulating its absorption and metabolism, so it is important to further investigate the mechanism of the combined intervention of the two on the alleviation of ulcerative colitis. Furthermore, there is a scarcity of studies examining the combined treatment of quercetin and lutein in the context of colitis alleviation. In this study, we utilized a mouse model of DSS-induced colitis to analyze tissue damage and serum inflammatory factor levels in the colons of the mice, thereby further investigating the alleviating effects of quercetin and lutein on colitis. Additionally, we analyzed the distribution characteristics of the intestinal microbiota in mice following gavage intervention using a second-generation sequencing platform to explore the combined effects of quercetin and lutein on colitis alleviation. This research aims to optimize the pharmacological activities of quercetin and lutein, offering a scientific foundation for the creation of functional products combining these two compounds.

## 2 Materials and methods

### 2.1 Materials and chemicals

C57BL/6 male mice were obtained from Beijing Spefo Co. (Beijing, China). Lutein (≥98%) and quercetin (≥98%) were purchased from Shanghai Yuan Ye. Sodium dextran sulfate (DSS), catalase (CAT), malondialdehyde (MDA), and myeloperoxidase (MPO) kits were sourced from Nanjing Jiancheng Bioengineering Institute (Nanjing, Jiangsu Province, China). Interferon γ (INFγ), interleukin 6 (IL-6), interleukin 1β (IL-1β), tumor necrosis factor α (TNF-α), and interleukin 10 (IL-10) were obtained from Shanghai Enzymotec Biotechnology Co. Tight junction proteins occludin (GB111401) and claudin (GB152543) were acquired from Servicebio (Wuhan, Hubei Province, China), while ZO-1 (21773-1-AP) was sourced from PTGLAB (Wuhan, Hubei Province, China). The RNA Easy Fast Animal Tissue Total RNA Extraction Kit, FastKing One-Step Degenomic cDNA First Strand Synthesis Premix Kit, and FastReal Rapid Fluorescence PCR Premix Kit (SYBR Green) were purchased from Tiangen Biochemical Technology Co. (Beijing, China).

### 2.2 Animal experiment

We affirm that all research procedures adhered to the appropriate ethical guidelines. Ethical approval for this study was obtained from the Animal Ethics Committee of Hebei Agricultural University, with reference number (2023197).

Sixty male C57BL/6 mice were selected for an acclimatization period with ad libitum access to diet and water. The acclimatization environment was maintained for 1 week. The mice were subsequently divided into a control group and a disease model group. Ten mice in the control group (NC group) received normal water, while the remaining mice were administered a daily dose of 3.5% W/VDSS for five consecutive days to induce acute colitis. Following the modeling, the mice in the disease model group were randomly assigned to five groups: the model group (IBD group), the lutein gavage group (LU group), the quercetin gavage group (QR group), the lutein and quercetin co-treatment group (LU + QR group), and the 5-aminosalicylic acid treatment group (5-ASA group). Each group consisted of 10 mice. Quercetin, lutein, and 5-aminosalicylic acid were dissolved in 0.5% sodium carboxymethyl cellulose. The final gavage doses were 20 mg/kg/bw for the lutein group, 100 mg/kg/day for the quercetin group, 100 mg/kg/bw of quercetin combined with 20 mg/kg/bw of lutein in the co-treatment group, and 50 mg/kg/bw of 5-aminosalicylic acid in the 5-ASA group. The model group received the same daily dose of sodium carboxymethyl cellulose. A total of 10 days of gavage were conducted, with each mouse receiving a gavage of 0.2 mL once daily. Observations of body weight, water intake, food intake, fecal viscosity, fecal occult blood, and overall mouse health were recorded throughout the modeling and gavage periods. At the conclusion of the gavage, the mice were fasted for 12 h. Whole blood was collected via ocular puncture to obtain serum. Tissue samples were harvested from cervical dislocated mice, with the colorectum excised from the cecum to the anus on clean cardboard, and the length of the colon was measured and documented. The liver and spleen were also excised, surface moisture was blotted with filter paper, and the organ weights were recorded to calculate the organ index. The collected tissues and serum were subsequently stored at −80 °C for future analysis.

### 2.3 Histopathological analysis of mouse colon, determination of the number of cup cells and immunohistochemical analysis

The collected colon tissues were fixed in 4% paraformaldehyde and trimmed to the appropriate size. The sections were subsequently embedded in paraffin solution, followed by deparaffinization in a deparaffinizing solution. Afterward, the tissues were soaked in ethanol and washed with tap water. The staining process involved sequential staining with hematoxylin dye, followed by eosin dye for the nuclei of the cells. The stained tissues were then dehydrated using ethanol and subjected to xylene immersion. Finally, the tissues were neutralized with neutral tree glue before sealing. After drying, the morphology of the colon tissue was observed using light microscopy.

Using a photomicrographic microscope, the intestinal gland region of the colon tissue was selected for imaging at 100× magnification. The imaging was conducted to maximize the coverage of the tissue in the field of view, ensuring consistent background lighting across all photographs. Following the imaging process, the length of the intestinal gland epithelium was measured at five locations within each section using image analysis software. Additionally, the number of cup cells within the intestinal gland epithelium was counted to calculate the density of cup cells per unit length, defined as the number of cup cells divided by the length of the intestinal gland epithelium.

The immunohistochemistry testing process begins with the identification of the tissue sample, where the area of interest is automatically located and circled. Manual adjustments can be made based on specific requirements. In terms of color selection, the HSI (hue, saturation, intensity brightness) system is employed for automatic positive judgment and classification of the positivity levels: weakly positive is indicated by a yellowish color (1 point), moderately positive by a brownish-yellow color (2 points), and strongly positive by a brown color (3 points). A positive tan color also corresponds to 3 points and can be manually adjusted as necessary. The software subsequently locates the cell nuclei and expands the cytoplasmic range as required. It calculates the number of weak, moderate, and strong positive cells along with their respective areas, as well as various parameters such as the integrated optical density (IOD) and tissue area and other different parameters. Finally, the area to be measured. The measurement area is calculated step by step at high magnification. Upon completion of the analysis, each parameter is automatically calculated based on the original data and algorithmic formulas. The average optical density value reflects the average intensity of the positive signal, and this value is used to evaluate the strength of the positive signal.

### 2.4 Determination of serum inflammatory factor levels in mice

Blood samples were collected from the eyeballs of mice and allowed to clot at room temperature for 30 min. The serum was then separated by centrifugation at 3,000 rpm for 10 min. The resulting supernatant (serum) was carefully aliquoted and stored at −80 °C until analysis to avoid repeated freeze–thaw cycles. Quantification of inflammatory cytokines (TNF-α, IL-1β, and IL-6) and oxidative stress markers (MPO, MDA, and SOD) was performed using specific commercial ELISA kits in strict accordance with the manufacturer’s instructions. Briefly, all reagents, samples, and standards were brought to room temperature and prepared as required. Then, standards and diluted serum samples were added to the antibody-precoated wells in quintuplicate. After the final step, the reaction was terminated using stop solution, and the optical density (OD) of each well was immediately measured at 450 nm using a microplate reader. A standard curve was plotted based on the known concentrations of the standards, and the concentrations of target analytes in the samples were calculated accordingly. All samples were assayed with five replicates, and the average values were used for statistical analysis.

### 2.5 Determination of inflammation-related gene expression profiles in mice colon tissue

Weigh the appropriate amount of mouse colon tissue and add RLA lysate. Homogenize the mixture using a high-throughput tissue grinder, then add 10 μL of proteinase K mix thoroughly and incubate at room temperature for 5 min. Centrifuge the homogenate at 12,000 rpm for 3 min, and proceed with total RNA extraction using the RNA Easy Fast Animal Tissue Total RNA Extraction Kit. The purity and concentration of the extracted RNA were assessed, with the OD260/OD280 ratio, an indicator of protein contamination, expected to fall between 1.8 and 2.1. The extracted RNA underwent reverse transcription, with the reaction was perform conducted at 42 °C for 15 min to eliminate genomic DNA, followed by a 95 °C incubation for 3 min to inactivate the enzyme. Appropriate primer sequences were identified and validated based on primer design principles via the NCBI website, and the primers were subsequently synthesized by General Biologicals. The Real-Time PCR reaction mixture was prepared on ice for quantitative fluorescence PCR, using β-actin as an internal reference. Relative quantification was calculated using the 2^−ΔΔCT^ method.

### 2.6 Extraction and determination of short-chain fatty acids

The contents of the cecum are mixed with five times the volume of deionized water and centrifuged for 10 min at 4 °C and 12,000 × g. The resulting supernatant is combined with an equal volume of 2-butylacetic acid. Subsequently, the mixture is filtered and analyzed using gas chromatography.

### 2.7 Determination of intestinal flora in mouse cecum contents

The contents of the mouse cecum were freeze-dried under vacuum and subsequently ground. The resulting material was centrifuged at 10,000 rpm for 30 min. Genomic DNA was isolated from the pellet using a DNA extraction kit. The concentration and purity of the isolated DNA were assessed using 1% agarose gel electrophoresis and a NanoDrop-2000 spectrometer. The V3–V4 region of the 16S rRNA gene was amplified via PCR, and the amplification results were verified using 2% agarose gel electrophoresis. The purified PCR products were then analyzed through high-throughput sequencing.

### 2.8 Data analysis

All experimental procedures were replicated a minimum of three times, with quantitative results presented as mean ± standard deviation. Statistical analyses were performed using SPSS software, where one-way ANOVA was applied to evaluate group differences, with a *p*-value <0.05 establishing statistical significance.

## 3 Results

### 3.1 Effects of different treatments on changes in body weight, DAI index, organ index, and colon length in mice

**Figure 1:**
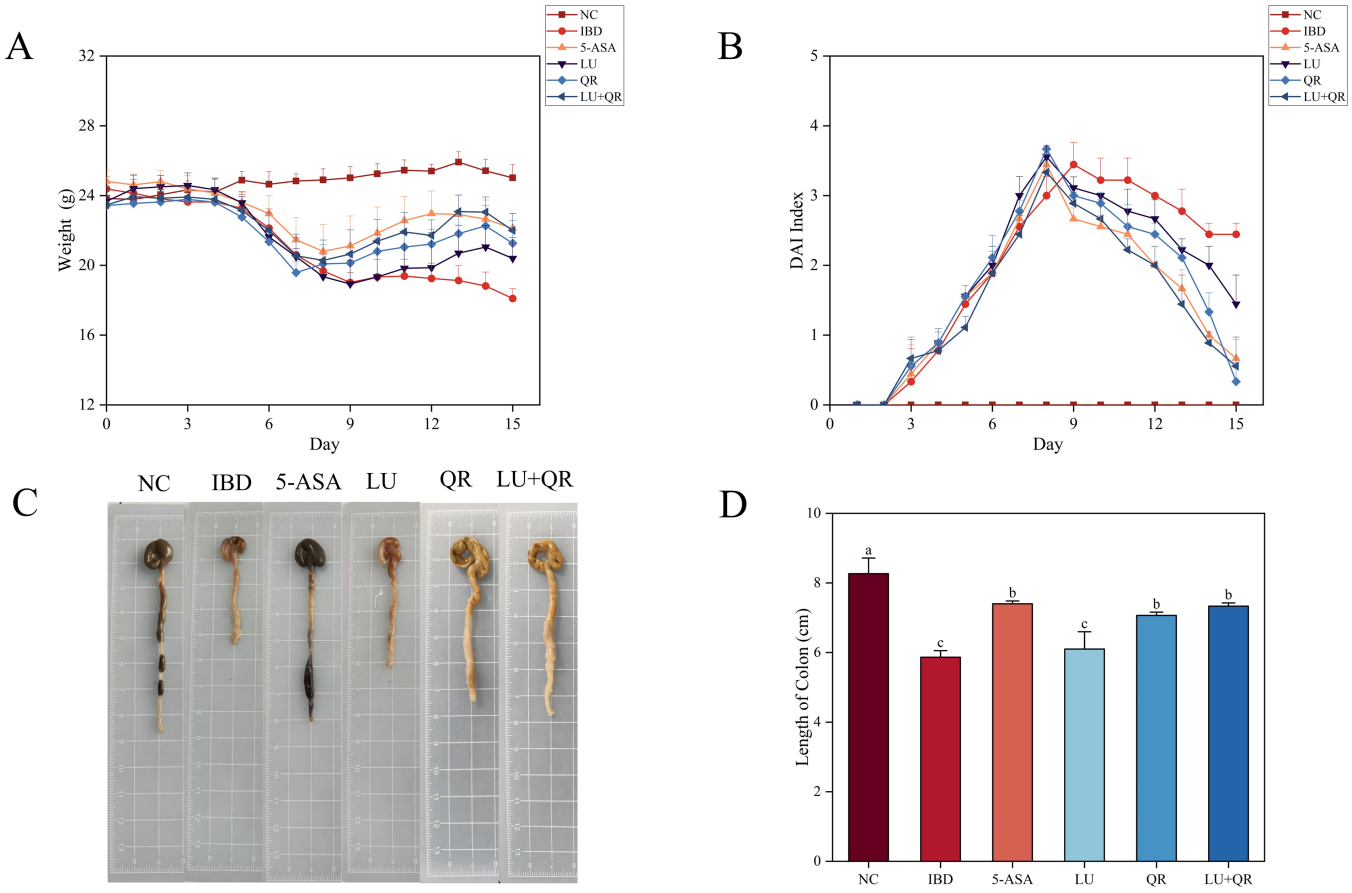
Effects of different treatments on body weight **(A)**, DAI index **(B)**, schematic length of colon tissue **(C)**, and length of colon **(D)** in mice. NC: blank control group; IBD: model group; 5-ASA: 5-aminosalicylic acid official gavage group; LU: lutein gavage group; QR: quercetin gavage group; LU + QR: quercetin and lutein combined gavage group. The error bar represents the standard deviation. Different letters indicate significant differences between groups (*p* < 0.05).

The body weight and disease activity index (DAI) profiles of mice were recorded during the modeling of DSS treatment and the subsequent drug administration. As illustrated in Figure 1A, the overall body weight of mice in the inflammatory bowel disease (IBD) group exhibited a decreasing trend. In contrast, the body weight of mice in the 5-ASA, LU, QR, and LU + QR groups initially decreased but subsequently increased, indicating a recovery in body weight post-treatment that approached that of the normal control (NC) group. This suggests an alleviation of colitis symptoms in the treated mice. The DAI index serves as a crucial tool for assessing the severity of colonic symptoms. Following administration of 3.5% DSS, the mice exhibited reduced water intake and dietary consumption, diarrhea, blood in the stools, and erect, lusterless fur. As shown in Figure 1B, throughout the testing period, the DAI index of the mice demonstrated an initial increase followed by a decrease, with the exception of the NC group. During the treatment period, the DAI indices of the treated groups increased more significantly compared to the IBD group, ultimately falling below 2 for all groups. The liver and spleen indices of mice serve as approximate indicators of immune function strength, while colon inflammation impacts the size of both the liver and spleen. As illustrated in Table 1, the liver and spleen indices of mice in the inflammatory bowel disease (IBD) group were significantly elevated compared to those in the normal control (NC) group, with notable splenomegaly observed. In contrast, the LU + QR group exhibited a reduction in spleen and liver indices by 33.3 and 22.3%, respectively, relative to the IBD group, thereby mitigating splenomegaly and hepatic enlargement induced by colon inflammation and improving colitis symptoms in the mice. Furthermore, the liver and spleen indices of the LU + QR group were lower than those of the NC group. Colon inflammation was also associated with a reduction in colon length. The colon images of mice in different groups are shown in Figure 1C. As depicted in Figure 1D, the IBD group experienced a significant reduction in colon length compared to the NC group, while the 5-ASA, QR, and LU + QR groups demonstrated a significant increase in colon length relative to the IBD group. These experimental results suggest that the combined treatment of lutein and quercetin can alleviate the shortening of the mouse colon, thereby reducing colon inflammation.

**Table 1:** Organ indices in mice.

Organ	NC	IBD	5-ASA	LU	QR	LU + QR
Spleen	0.32 ± 0.02^d^	0.63 ± 0.04^a^	0.41 ± 0.02^c^	0.57 ± 0.05^b^	0.47 ± 0.03^c^	0.42 ± 0.03^ab^
Liver	3.86 ± 0.13^b^	4.96 ± 0.49^a^	4.00 ± 0.20^b^	4.76 ± 0.36^a^	4.05 ± 0.44^b^	3.85 ± 0.24^b^

Values are expressed as the mean ± S.D. The different letters in same row indicate the significant different (*p* < 0.05).

### 3.2 Histopathologic analysis of mice and changes in the number of cup cells

**Figure 2:**
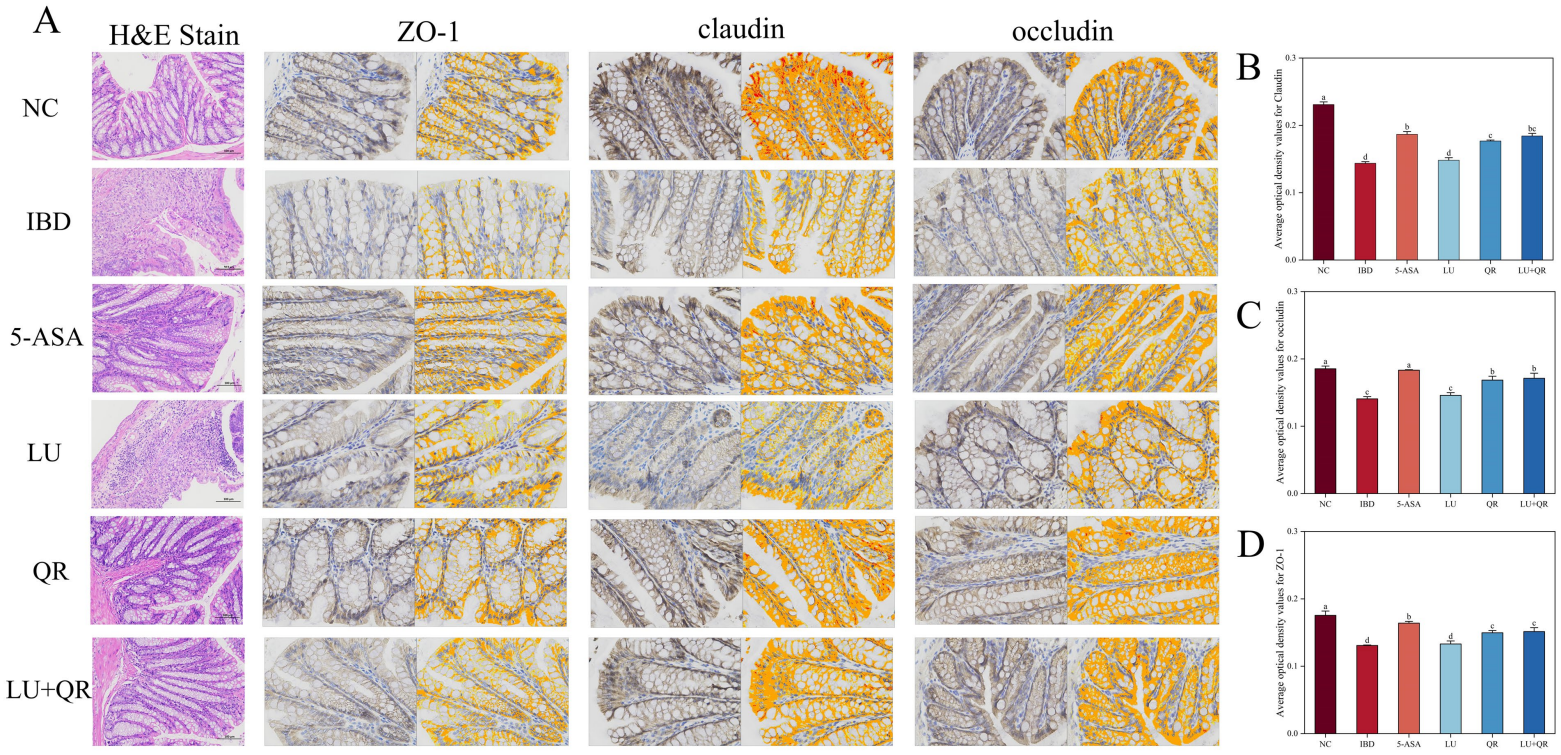
Histopathologic staining and scoring of mouse colon. **(A)** H&E staining and immunohistochemical analysis of mouse colon tissue. Changes in expression of tightly linked proteins claudin **(B)**, occludin **(C)**, and ZO-1 **(D)**. NC: blank control group; IBD: model group; 5-ASA: 5-aminosalicylic acid official gavage group; LU: lutein gavage group; QR: quercetin gavage group; LU + QR: quercetin and lutein combined gavage group. The error bar represents the standard deviation. Different letters indicate significant differences between groups (*p* < 0.05).

**Figure 3:**
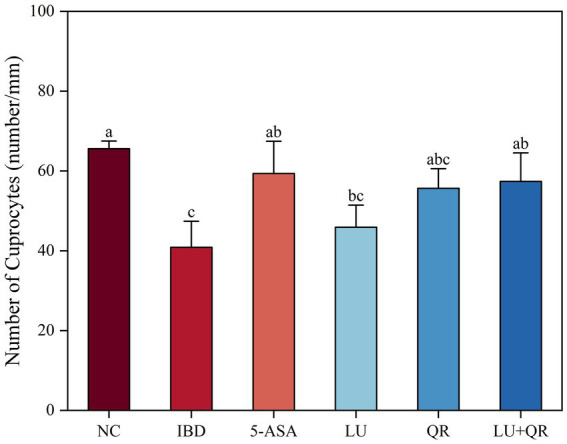
Effect of different treatments on changes in the number of cup cells in mouse colon tissue. NC: blank control group; IBD: model group; 5-ASA: 5-aminosalicylic acid official gavage group; LU: lutein gavage group; QR: quercetin gavage group; LU + QR: quercetin and lutein combined gavage group. The error bar represents the standard deviation. Different letters indicate significant differences between groups (*p* < 0.05).

The colonic tissues of mice were examined using hematoxylin–eosin staining, as illustrated in Figure 2A. In the NC group, the mucosal layer of the colonic tissues protruded into the intestinal lumen, forming folds. Numerous intestinal glands were densely arranged in the lamina propria, with a significant presence of cup-shaped cells. The structure of the submucosal muscle layer was well-defined. Conversely, in the model group, disorganized colon tissue was observed, characterized by large ulcers, destruction of the intestinal gland structure and mucosal epithelium, increased infiltration of lymphocytes and granulocytes in the lamina propria and submucosa, and irregularly shaped intestinal glands. Following drug treatment, the ulcers exhibited a reduction in size, while lymphocyte and granulocyte infiltration decreased, leading to a clearer structure of the muscularis propria. This suggests that the symptoms of colitis in the treated group of mice were alleviated compared to the model group. Furthermore, intestinal epithelial tight junction proteins are responsible for controlling the permeability of the intestinal mucosa and play a crucial role in maintaining the intestinal barrier. Therefore, the expression of tight junction proteins in mouse colon tissues was assessed. As illustrated in Figure 2, the expression levels of ZO-1, claudin, and occludin proteins were markedly decreased within the colon tissues of mice treated with DSS compared to the NC group. In contrast, treatment with 5-ASA, QR, and LU + QR resulted in a significant increase in the expression of tight junction proteins, while no significant difference was observed in the LU group. These experimental results indicate that the drug-treated groups facilitated the repair of the gut mucosal barrier by enhancing the levels of TJ (tight junction) proteins, thereby alleviating the symptoms of colitis in mice. Furthermore, cuprocytes play a crucial role in synthesizing and secreting mucin, which forms a mucosal barrier to protect epithelial cells. As depicted in Figure 3, the quantification of cuprocytes in mouse colon tissue revealed a significant decrease in the model group compared to the control group. Conversely, the number of cuprocytes in the drug-treated LU + QR and 5-ASA groups was considerably higher than that in the IBD group, with increases of 27 and 33.8%, respectively.

### 3.3 Changes of serum inflammatory factors in mice

**Figure 4:**
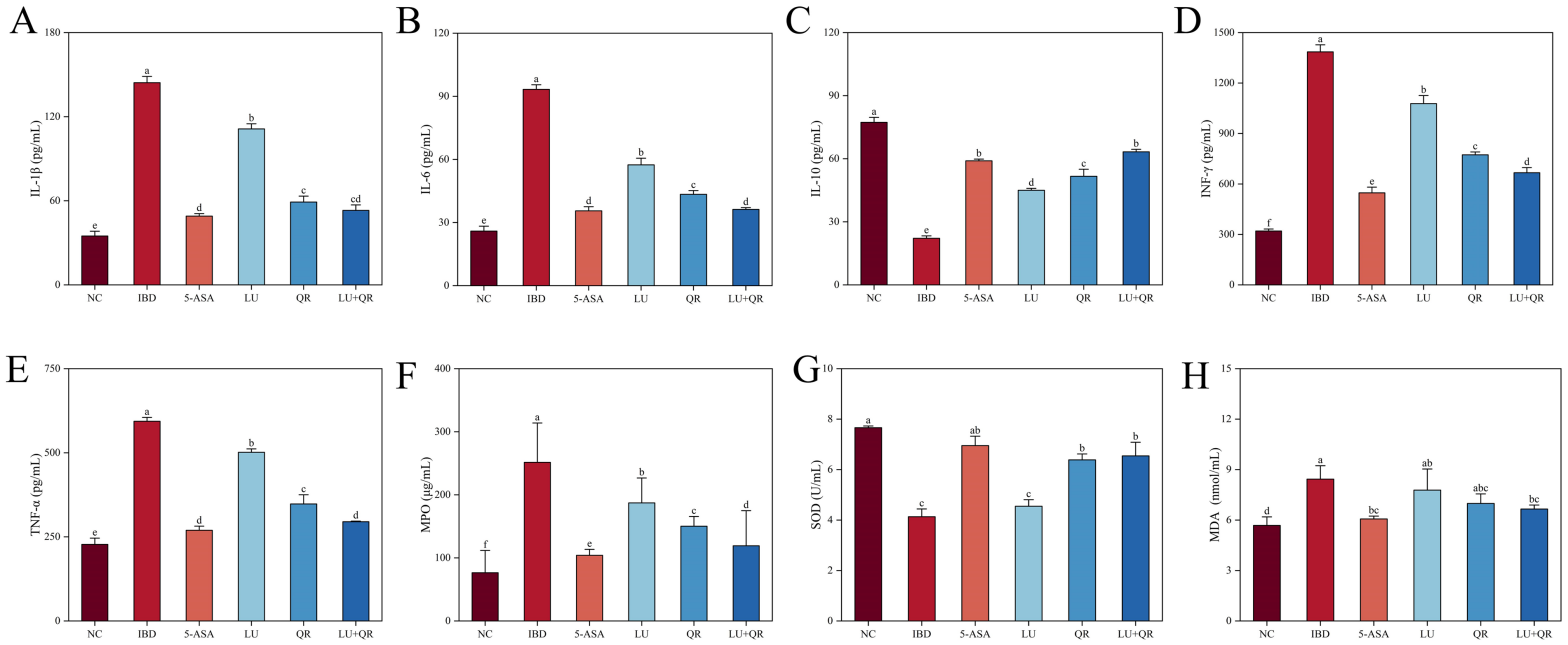
Effects of different treatments on serum levels interleukin of 1β (IL-1β) **(A)**, interleukin 6 (IL-6) **(B)**, interleukin 10 (IL-10) **(C)**, interferon-gamma (INF-γ) **(D)**, and tumor necrosis factor-alpha (TNF-α) **(E)**, myeloperoxidase (MPO) **(F)**, superoxide dismutase (SOD) **(G)**, malondialdehyde (MDA) **(H)**. NC: blank control group; IBD: model group; 5-ASA: 5-aminosalicylic acid official gavage group; LU: lutein gavage group; QR: quercetin gavage group; LU + QR: quercetin and lutein combined gavage group. The error bar represents the standard deviation. Different letters indicate significant differences between groups (*p* < 0.05).

As demonstrated in Figure 4, treatment with DSS led to a substantial rise in the levels of IL-1β, IL-6, INF-γ, and TNF-α in the colonic tissues of mice compared to the NC group, while the level of IL-10 decreased significantly. All therapeutic interventions demonstrated significant downregulation of key pro-inflammatory mediators (IL-1β, IL-6, INF-γ, TNF-α) compared to untreated IBD controls, with the LU + QR combinatorial treatment and 5-ASA monotherapy achieving superior anti-inflammatory efficacy. Additionally, the treated groups significantly increased the levels of IL-10 compared to the IBD group. The test results indicated that the combination of quercetin and lutein could ameliorate colonic inflammation by reducing anti-inflammatory factors and enhancing pro-inflammatory factors. Following DSS treatment, the oxidative stress indices in the mice demonstrated significant abnormalities compared to the NC group. Specifically, the levels of MPO and MDA increased significantly, while the level of SOD decreased significantly. As illustrated in Figures 4F,H, the MPO activity and MDA levels in the treated groups were lower than those in the IBD group, with the LU + QR group showing reductions of 52.6 and 20.2%, respectively. Furthermore, as shown in Figure 4G, the SOD activity in the QR, LU + QR, and 5-ASA groups significantly increased compared to the IBD group. These experimental results suggest that the treatment groups normalized oxidative stress levels and mitigated oxidative damage caused by colitis, thereby enhancing the antioxidant capacity in the colon.

### 3.4 The expression levels of inflammation-related genes in mice colon tissue

**Figure 5:**
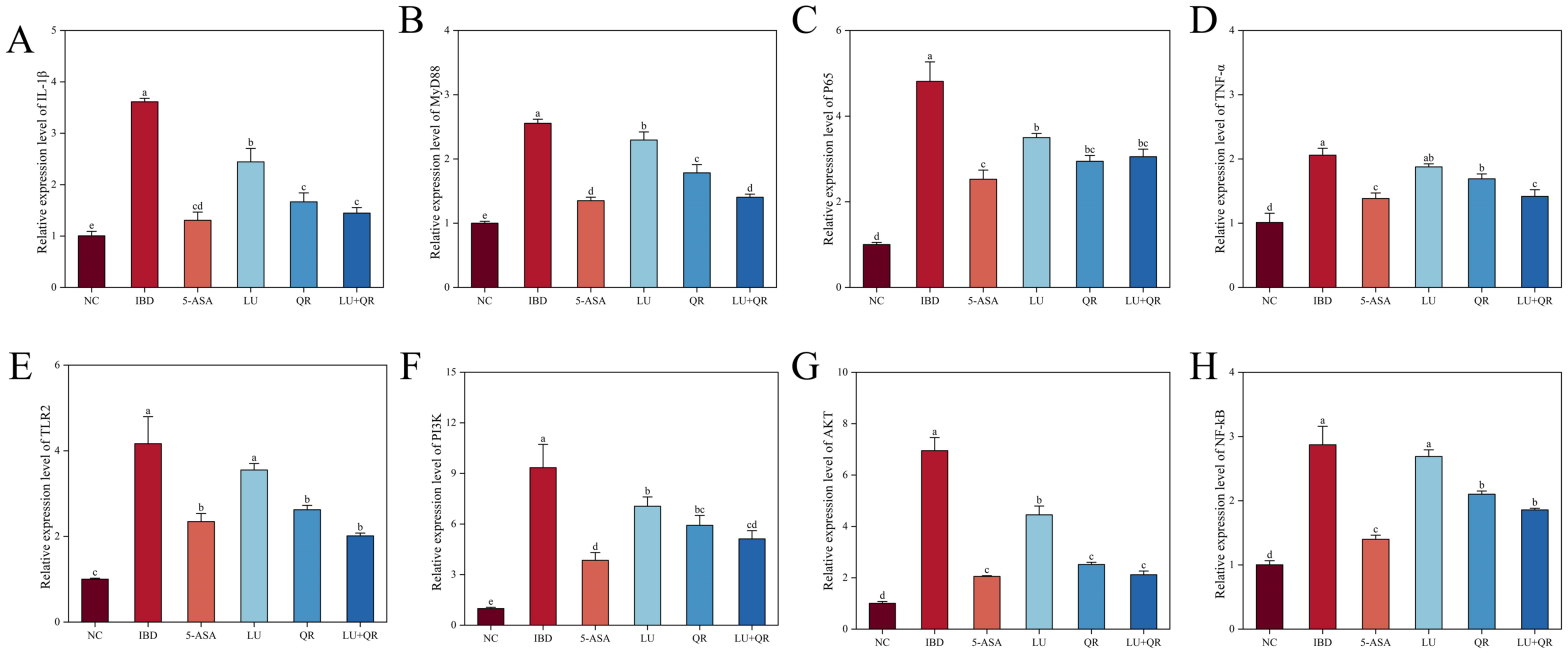
Relative expression of inflammation-related genes IL-1β **(A)**, MyD88 **(B)**, P65 **(C)**, TNF-α **(D)**, TLR2 **(E)**, PI3K **(F)**, AKT **(G)**, and NF-kB **(H)** in mouse colon tissues. NC: blank control group; IBD: model group; 5-ASA: 5-aminosalicylic acid official gavage group; LU: lutein gavage group; QR: quercetin gavage group; LU + QR: quercetin and lutein combined gavage group. The error bar represents the standard deviation. Different letters indicate significant differences between groups (*p* < 0.05).

The mRNA expression levels of IL-1β, MyD88, P65, TNF-α, TLR2, PI3K, AKT, and NF-κB genes in mouse colon tissues were assessed using quantitative PCR (qPCR). As illustrated in Figure 5, DSS treatment resulted in a significant increase in the mRNA expression levels of IL-1β, MyD88, P65, TNF-α, TLR2, PI3K, AKT, and NF-κB. In comparison to the IBD group, the LU + QR group exhibited a notable reduction in the mRNA expression levels of these genes in colon tissues. Specifically, as depicted in Figure 5G, the expression level of AKT mRNA decreased by 69.5% in the LU + QR group relative to the IBD group, representing the most substantial improvement among the assessed gene expression levels.

### 3.5 Determination of short-chain fatty acids in mouse

Short-chain fatty acids (SCFAs) play crucial roles in maintaining intestinal health, including anti-inflammatory effects, preservation of intestinal barrier function, and modulation of immune responses. In this study, several common SCFAs—namely acetic acid, propionic acid, n-butyric acid, isobutyric acid, n-valeric acid, and isovaleric acid—were quantified in mouse colon tissues. As presented in Table 2, the concentrations of each SCFA in the colonic tissues of mice treated with dextran sulfate sodium (DSS) exhibited a significant decrease. The 5-ASA, LU, QR, and LU + QR treatment groups demonstrated varying degrees of increase in SCFA levels compared to the inflammatory bowel disease (IBD) group, approaching the levels observed in the normal control (NC) group. Notably, the total SCFA content in the LU + QR group increased by 77.07% relative to the IBD group. These findings indicate that quercetin and lutein can effectively regulate SCFA levels in the intestine.

**Table 2:** Short-chain fatty acid content of the cecum.

SCFA (μg/g)	NC	IBD	5-ASA	LU	QR	LU + QR
Acetic acid	674.39 ± 4.4^a^	362 ± 102.74^cd^	505.72 ± 34.82^b^	311.72 ± 11.66^d^	465.25 ± 1.31^bc^	525.72 ± 63.82^b^
Propanoic acid	2041.56 ± 162.67^a^	956.52 ± 25.43^d^	1378.06 ± 76.86^b^	1219.26 ± 50.88^bc^	2041.15 ± 18.01^a^	2211.5 ± 191.6^a^
Isobutyric acid	233.61 ± 1.88^a^	78.83 ± 0.96^d^	199.55 ± 11.26^b^	128.14 ± 11.51^c^	143.69 ± 18.55^c^	183.6 ± 11.57^b^
n-Butyric acid	3713.88 ± 269.83^a^	1749.94 ± 29.32^d^	2629.31 ± 74.38^b^	2163.42 ± 58.26^c^	2515.02 ± 18.54^b^	2563.09 ± 150.31^b^
Isovaleric acid	274.82 ± 23.59^a^	99.93 ± 1.66^d^	251.87 ± 6.76^ab^	163.16 ± 5.65^c^	233.42 ± 14.78^b^	238.36 ± 6.07^b^
Valeric acid	232.44 ± 58.79^a^	36.69 ± 8.45^b^	177.53 ± 2.84^a^	39.02 ± 2.14^b^	41.09 ± 1.64^b^	92.7 ± 12.85^b^
Total SCFAs	7170.69 ± 458.18^a^	3283.92 ± 61.57^e^	5142.03 ± 181.94^c^	4024.73 ± 92.66^d^	5439.63 ± 27.07^bc^	5814.97 ± 230.29^b^

Values are expressed as the mean ± S.D. The different letters in same row indicate the significant different (*p* < 0.05).

### 3.6 Effect of different treatments on the abundance of intestinal flora communities in the contents of mouse cecum

**Figure 6:**
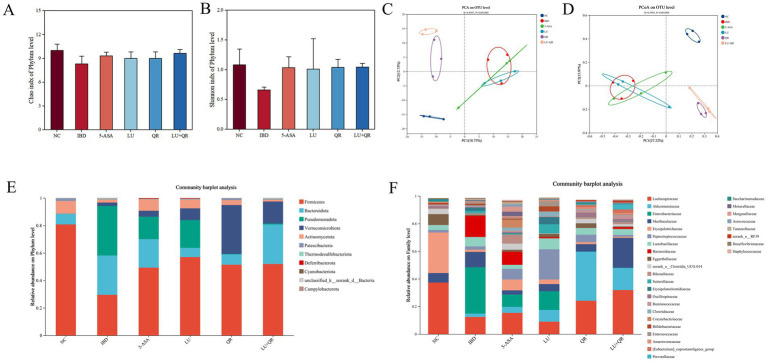
Effect of different treatments on the diversity of intestinal flora in mice. Chao index **(A)**, Shannon index **(B)**, PCA analysis **(C)**, PCoA analysis **(D)**, flora composition at the phylum level. **(E)**, flora composition at the family level **(F)**. NC: blank control group; IBD: model group; 5-ASA: 5-aminosalicylic acid official gavage group; LU: lutein gavage group; QR: quercetin gavage group; LU + QR: quercetin and lutein combined gavage group. The error bar represents the standard deviation. Different letters indicate significant differences between groups (*p* < 0.05).

The Chao index is a metric used to evaluate species richness, specifically measuring the total number of microbial species present in a sample. A higher Chao index signifies greater species richness. As illustrated in Figure 6A, the Chao index in mice decreased following DSS treatment, suggesting a reduction in the diversity of intestinal flora or a homogenization of the microbial community. Conversely, the Chao index increased in the LU + QR and 5-ASA groups, indicating a restoration of species richness within the flora. The Shannon index, which reflects species diversity, is depicted in Figure 6B. The Shannon index for the IBD group decreased, potentially due to an imbalance in flora and the onset of intestinal inflammation. After treatment, the Shannon index for species in the IBD group increased, with a notable 59.1% rise in the LU + QR group. The results of PCA analysis of β-diversity are presented in Figure 6C, while the PCoA analysis of β-diversity is shown in Figure 6D. These analyses revealed a significant alteration in the intestinal flora structure of mice in the IBD group compared to the NC group. Following treatment with all drug groups, the flora structure in the IBD group approached that of the NC group, with the most pronounced effect observed in the LU + QR group. This indicates that both quercetin and lutein possess the ability to regulate DSS-induced alterations in the intestinal flora of mice. The species composition at the phylum level is illustrated in Figure 6E, with the predominant phyla being Firmicutes, Bacteroidota, and Pseudomonadota. In the IBD group, the relative abundance of Firmicutes decreased, while the relative abundance of Bacteroidota and Pseudomonadota increased, resulting in a decreased F/B ratio. Following treatment, the relative abundance of Firmicutes increased in the treatment group, whereas the relative abundance of Pseudomonadota significantly decreased in the QR and LU + QR groups. As illustrated in Figure 6F, the composition of the intestinal flora in mice was analyzed at the genus level, revealing that *Lachnospiraceae*, a member of Firmicutes, had the highest relative abundance. Previous studies have demonstrated that the occurrence of colitis correlates with a reduction in the relative abundance of *Lachnospiraceae*, which aligns with the findings of the present study. Additionally, the results indicated a significant increase in the relative abundance of *Enterorhabdus*, a member of the phylum Aspergillus, while *Akkermansia* exhibited a decrease in abundance within the IBD group, potentially due to the disruption of the mucus layer in the mice. Notably, the relative abundance of *Enterorhabdus* decreased significantly following treatment in the treatment group. These experimental results suggest that quercetin and lutein ameliorated colitis in mice by modulating the composition of the intestinal flora.

## 4 Discussion

Current pharmacological treatments for colitis are often associated with significant side effects, highlighting the urgent need to develop natural compounds for both treatment and prevention. In this study, we utilized a DSS-induced ulcerative colitis animal model to investigate the effects of lutein and quercetin administered individually and in combination via gavage. Compared to the model group, the treatments significantly ameliorated colitis symptoms in mice, as evidenced by increased body weight, restored colon length, reduced pro-inflammatory factor levels, and enhanced expression of tight junction proteins. Previous studies have indicated that quercetin may improve the bioavailability of lutein, as its hydrophilic nature complements the lipophilicity of lutein. Furthermore, quercetin may modulate lutein bioavailability by regulating lutein transporter proteins and related enzymatic activities. Additionally, the inhibitory effect of quercetin on pro-inflammatory cytokines may enhance the antioxidant properties of lutein (17). Thus, the combination of quercetin and lutein may produce synergistic anti-inflammatory effects.

Observation of signs and symptoms in mice is a critical aspect of colitis assessment, encompassing various parameters such as body weight, food intake, water consumption, fecal consistency, overall condition, and response to inflammatory bowel disease severity (18). Our results demonstrate that both quercetin and lutein alone alleviated murine colitis symptoms, while their combined treatment showed more pronounced effects in reducing the disease activity index score and increasing colon length, comparable to the positive control drug 5-aminosalicylic acid. Histopathological analysis revealed a significant increase in the number of goblet cells in colonic tissues of the co-treatment group compared to the model group. Goblet cells secrete mucins that not only protect the intestinal epithelium from microorganisms and invading pathogens but also provide a suitable environment for commensal bacteria. Conversely, intestinal microbiota dysbiosis can lead to mucin dysfunction, enabling other commensals and their metabolites to cross the intestinal epithelium and potentially trigger host immune responses (19). Quercetin may exert a protective effect on the intestinal mucosal barrier by modulating the secretory function of intestinal goblet cells and mucin levels in enterocytes (20). The intestinal mucosal barrier is an innate defense mechanism that maintains intestinal homeostasis and impedes pathogenic bacteria and toxins, with tight junctions being central to its structure (21). Deficiency of tight junction proteins in the intestinal epithelium results in increased intestinal permeability, thereby elevating susceptibility to pathogenic bacterial invasion and excessive release of inflammatory factors in the gut, ultimately leading to intestinal inflammation (22). The expression levels of tight junction proteins serve as critical biomarkers for evaluating therapeutic interventions in colitis. In this study, DSS-treated mice exhibited downregulated expression of ZO-1, claudin-1, and occludin in colonic tissues, whereas LU + QR administration significantly upregulated the expression of these proteins, effectively restoring intestinal barrier integrity.

The dynamic balance between pro-inflammatory and anti-inflammatory mediators determines the magnitude of inflammatory responses, making their relative concentrations quantifiable biomarkers for assessing inflammatory status. Quercetin and lutein synergistically alleviate inflammation by targeting NF-κB and MAPK pathways, collectively suppressing the production of pro-inflammatory mediators (TNF-α, IL-6, IL-1β) at both transcriptional and secretory levels (23).

NF-κB is a key pro-inflammatory transcription factor that plays a critical role in the transcriptional activation of inflammatory factors such as TNF-α, IL-6, and IL-1β (24). In this study, we measured the mRNA expression levels of inflammatory factors IL-1β, MyD88, P65, TNF-α, TLR2, PI3K, AKT, and NF-κB within the PI3K/AKT/NF-κB signaling pathway. The results indicated that these inflammatory factors were upregulated after DSS treatment and downregulated following LU + QR intervention. It has been suggested that the PI3K/AKT signaling pathway may act as an upstream activator of the NF-κB signaling pathway. Zha et al. demonstrated that flavonoid soybean saponin may regulate NF-κB signaling by inhibiting the PI3K/AKT-mediated pathway, thereby suppressing inflammatory responses (25). Moreover, flavonoids can scavenge ROS by upregulating superoxide dismutase (SOD), a crucial antioxidant enzyme. Since ROS can activate various intracellular signaling pathways, including MAPK (p38, JNK, and ERK1/2), PKC, NF-κB, and PI3K/Akt, the ability of flavonoids to enhance SOD activity suggests their potential to modulate these signaling pathways. Carotenoids have also been shown to inhibit NF-κB signaling, which is significant for reducing inflammation and preventing its progression (26). Flavonoids exhibit the potential to suppress ROS generation by enhancing SOD activity, thereby regulating associated signaling cascades. In parallel, carotenoids act as potent inhibitors of NF-κB signaling, serving as critical regulators in attenuating inflammatory responses and preventing their pathological progression (27).

Short-chain fatty acids (SCFAs) are metabolites produced through the fermentation of dietary fibers by gut microbiota. Yukihiro et al. demonstrated that T cells play a pivotal role in suppressing inflammation and allergic responses, and that butyric acid, a product of intestinal microbial fermentation, promotes T cell differentiation, thereby alleviating intestinal inflammation. Additionally, it has been established that butyric acid can inhibit NF-κB activation by suppressing deacetylase activity (28). In the present study, we observed a decrease in short-chain fatty acid levels in the intestines of mice treated with DSS, while an increase in short-chain fatty acid levels was noted in the treated group following intervention.

Alterations in the composition of the gut microbiota, along with an increase in pathogenic bacteria abundance, lead to intestinal homeostasis imbalance, potentially resulting in intestinal diseases (9). The diversity and abundance of gut microbial species are significantly reduced in colitis mice. Our study demonstrated a notable decrease in both specie richness and diversity of intestinal flora in mice following DSS induction. Furthermore, the LU + QR treatment restored species richness and diversity of intestinal flora in these mice. Compared to the NC group, the abundance of Firmicutes was reduced in the IBD group. Firmicutes include several genera that produce SCFAs, such as butyric acid (e.g., *Roseburia* spp., *Clostridium* spp.). The decrease in Firmicutes may lead to a deficiency of anti-inflammatory substances like butyric acid, thereby exacerbating inflammation (29). SCFAs can inhibit bacterial translocation by activating GPR41 and GPR43 receptors, thereby enhancing the intestinal barrier and preventing bacterial invasion of tissues (30). In this study, the abundance of Firmicutes increased in all treated groups after intervention. The increase in Bacteroidota abundance resulted in a decreased F/B (Firmicutes/Bacteroidota) ratio, which has been associated with increased intestinal permeability and inflammatory responses (31). In our study, the abundance of Bacteroidota decreased in the 5-ASA, LU, and QR groups. Elevated levels of pathogenic bacteria in the gut microbiota release lipopolysaccharide (LPS), which activates the TLR4/NF-κB pathway, driving inflammation (32). The highest percentage of Pseudomonadota abundance was observed in the IBD group. Additionally, it has been demonstrated that lutein promotes the proliferation of the mucus-degrading bacterium Akkermansia, thereby enhancing the protective mucus layer (33).

The combined intervention of quercetin and lutein demonstrated superior efficacy compared to monotherapy. Quercetin, a potent flavonoid antioxidant, directly scavenges reactive oxygen species (ROS) and enhances endogenous antioxidant defenses by upregulating enzymes such as glutamate-cysteine ligase (GCL) (34). Meanwhile, lutein, a lipid-soluble carotenoid, incorporates into cell membranes, providing structural protection against lipid peroxidation and enhancing barrier integrity (35). The combined application of both compounds may mitigate oxidative stress more comprehensively across both aqueous and lipid cellular compartments. Furthermore, the well-established role of quercetin in inhibiting the NF-κB pathway may create an anti-inflammatory environment that potentiates the barrier-strengthening effects of lutein. This multi-target strategy—simultaneously suppressing inflammation, restoring epithelial barrier function, and modulating the gut microbiota—proves particularly advantageous in alleviating complex diseases such as ulcerative colitis, which involves multiple intertwined pathological pathways. The observed restructuring of the gut microbiota, particularly the modulation of Firmicutes and Bacteroidota, is critical, as dysbiosis is a hallmark of ulcerative colitis. The combined intervention may foster a more favorable microbial environment, further promoting immune homeostasis and barrier protection (36). The quercetin-lutein combination represents a highly promising nutritional strategy for the management of colitis.

## 5 Conclusion

The combined treatment of quercetin and lutein demonstrated significant therapeutic efficacy in alleviating DSS-induced colitis in mice, surpassing the effects of monotherapy with either compound alone. The enhanced benefit is largely attributable to a multi-target synergistic mechanism. Quercetin improves the bioavailability of lutein through its hydrophilic properties, which complement the lipophilicity of lutein. Furthermore, the combination potently inhibits the NF-κB signaling pathway, leading to reduced expression and secretion of pro-inflammatory cytokines such as IL-1β, TNF-α, MyD88, TLR2, and p65. Concurrently, it upregulates intestinal tight junction proteins including ZO-1, claudin, and occludin, thereby restoring epithelial barrier integrity. Additionally, the co-administration modulates the disturbed gut microbiota by normalizing the relative abundances of Firmicutes, Bacteroidota, and Pseudomonadota. Collectively, these actions—enhancing barrier function, suppressing inflammation, and correcting dysbiosis—underline the potential of quercetin and lutein as a synergistic strategy for colitis intervention.

## Data Availability

The original contributions presented in the study are publicly available. This data can be found in the NCBI repository under accession number: PRJNA1344460.
